# A Multielement Ionomic Dysregulation Pattern Is Associated with NT-proBNP and Cardiorenal Burden in Patients with Cardiovascular Disease

**DOI:** 10.3390/molecules31203588

**Published:** 2026-10-09

**Authors:** Minseung Kang, Siwon Baek, Giuseppe Maria Raffa, Calogera Pisano, Vincenzo Nuzzi, Mariusz Kowalewski, Beata Krasińska, Piotr Suwalski, Yvgen Spasenenko, Antonio Segreto, Katarzyna Gabriel, Andrzej Tykarski, Zbigniew Krasiński, Julia Frąckowiak, Marek Jemielity, Tomasz Urbanowicz, Anetta Hanć

**Affiliations:** 1 College of Medicine, Korea University, 73 Goryeodae-ro, Seongbuk-gu, Seoul 02841, Republic of Korea; 2 College of Medicine, Inje University, 75 Bokji-ro, Busanjin-gu, Busan 47392, Republic of Korea; 3 Department of Precision Medicine in Medical, Surgical and Critical Area (Me.Pre.C.C.), University of Palermo, 90134 Palermo, Italy; 4 Department of Research, IRCCS ISMETT, 90127 Palermo, Italy; 5 Thoracic Research Center, Collegium Medicum, Nicolaus Copernicus University, 85-067 Bydgoszcz, Poland; 6 Department of Cardiac Surgery, Center of Postgraduate Medical Education, Central Clinical Hospital of the Ministry of Interior, 02-507 Warszawa, Poland; 7 Department of Hypertensiology, Angiology, and Internal Medicine, Poznan University of Medical Sciences, ½ Dluga Street, 61-848 Poznan, Poland; 8 Department of Vascular, Endovascular Surgery, Angiology and Phlebology, Poznan University of Medical Sciences, ½ Dluga Street, 61-848 Poznan, Poland; 9 Department of Trace Analysis, Faculty of Chemistry, Adam Mickiewicz University, 61-614 Poznan, Poland; 10 Cardiac Surgery and Transplantology Department, Poznan University of Medical Sciences, ½ Długa, 61-848 Poznan, Poland

**Keywords:** ionomics, trace elements, NT-proBNP, cardiorenal syndrome, congestion, heart failure, copper, strontium, rubidium, iron

## Abstract

Background: Cardiovascular hemodynamic stress may be associated with alterations in the circulating ionome. We investigated whether a hypothesis-driven multielement composite, the congestion-associated ionomic dysregulation score (CIDS; higher copper and strontium and lower rubidium and iron), was associated with N-terminal pro-B-type natriuretic peptide (NT-proBNP) and cardiorenal–hematologic burden. Methods: This retrospective, single-center, cross-sectional study included 192 patients undergoing cardiovascular evaluation or treatment. Serum trace elements were quantified by inductively coupled plasma mass spectrometry, and CIDS was calculated from standardized element concentrations. Results: Higher CIDS was associated with higher NT-proBNP (ρ = 0.416, FDR-adjusted *p* < 0.001), worse New York Heart Association (NYHA) functional status (ρ = 0.370, *p* < 0.001), lower hemoglobin (ρ = −0.287, *p* < 0.001), lower eGFR (ρ = −0.191, *p* = 0.012), and higher uric acid (ρ = 0.241, *p* = 0.002). In the common complete-case sample, each 1-SD increase in CIDS was associated with higher log-transformed NT-proBNP after multivariable adjustment (β = 0.276, 95% CI 0.077–0.475; HC3-robust *p* = 0.007), and model R^2^ increased from 0.368 to 0.407. The observed ΔR^2^ exceeded the median from randomly weighted composites but did not clearly exceed the permutation null distribution (empirical *p* = 0.063). The referral-stratified estimates differed in magnitude (β = 0.179 versus 0.569), without a significant interaction (*p* = 0.171). Conclusions: CIDS was associated with myocardial stress and cardiorenal–hematologic burden in this cohort, but the single-center, cross-sectional findings do not establish causality, unique superiority over alternative composites, or external biomarker validity. Because CIDS was standardized internally within this cohort, no externally applicable reference values or clinical cutoffs can currently be defined.

## 1. Introduction

The circulating ionome represents an integrated biochemical phenotype shaped by nutritional exposure, renal handling, protein binding, erythrocyte distribution, inflammation, oxidative stress, and tissue turnover [1,2,3]. Ionomics and elemental metabolomics use simultaneous multielement profiling to study relationships among elemental composition, physiological state, and biological phenotype rather than treating each element as an isolated exposure [4,5]. In cardiovascular disease, several of these processes may change concurrently, providing a rationale for evaluating multielement patterns alongside individual analytes.

Cardiac congestion is a systemic pathophysiologic state that encompasses more than elevated intracardiac filling pressures [6,7]. It is frequently accompanied by renal dysfunction, altered hepatic and inflammatory signaling, hemodilution or anemia-related changes, and metabolic abnormalities. N-terminal pro-B-type natriuretic peptide (NT-proBNP) is widely used as a clinical marker of myocardial wall stress and hemodynamic burden [6,8]; however, it does not, by itself, characterize the broader biochemical disturbances that accompany congestion [7].

Trace elements participate in oxygen transport, mitochondrial metabolism, antioxidant defense, inflammatory signaling, and cellular ion homeostasis [2,3,9]. Previous studies have reported altered circulating copper concentrations in acute and chronic heart failure, as well as associations between copper and NT-proBNP [10,11,12,13,14,15,16]. Iron deficiency and anemia are also common in heart failure and contribute to impaired oxygen delivery and cellular energetics [17,18,19,20,21,22]. Strontium and rubidium have been studied less extensively in cardiovascular disease, although their circulating concentrations may be influenced by renal handling, bone-mineral metabolism, intracellular distribution, and dialysis-related processes [3,23,24,25,26,27].

Most prior cardiovascular investigations have evaluated individual elements separately. This approach may miss joint variation arising from renal filtration, inflammation, hepatic function, nutritional status, erythrocyte mass, and tissue redistribution. Ionomic and metallomic methods instead characterize multiple elements within a shared analytical framework, commonly using ICP-MS for sensitive multielement measurement [10,11]. Recent ICP-MS-based metallomic studies further demonstrate the utility of combining multielement profiling with multivariate and chemometric approaches to identify disease-associated elemental patterns [28,29]. A composite score can summarize a specified directional pattern, but its performance and biological interpretation require comparison with individual elements and alternative composite specifications.

To our knowledge, the Cu-high/Sr-high/Rb-low/Fe-low pattern has not previously been evaluated as a composite index in a cardiovascular cohort. Because individual element–outcome associations may be modest, we examined whether this hypothesis-driven four-element summary was associated with NT-proBNP and related clinical measures. The analysis was intended to evaluate an exploratory composite rather than to establish a diagnostic biomarker or a uniquely coordinated biological mechanism.

We defined the congestion-associated ionomic dysregulation score (CIDS) as relatively higher concentrations of copper and strontium and lower concentrations of rubidium and iron. The components were selected to represent potentially relevant biological domains, but the proposed links to inflammatory, hepatic, renal, hematologic, and metabolic processes remain mechanistic hypotheses rather than effects demonstrated by the present cross-sectional data. We hypothesized that higher CIDS would be associated with higher NT-proBNP and would coexist with lower hemoglobin, reduced renal function, and higher uric acid.

**Figure 1:**
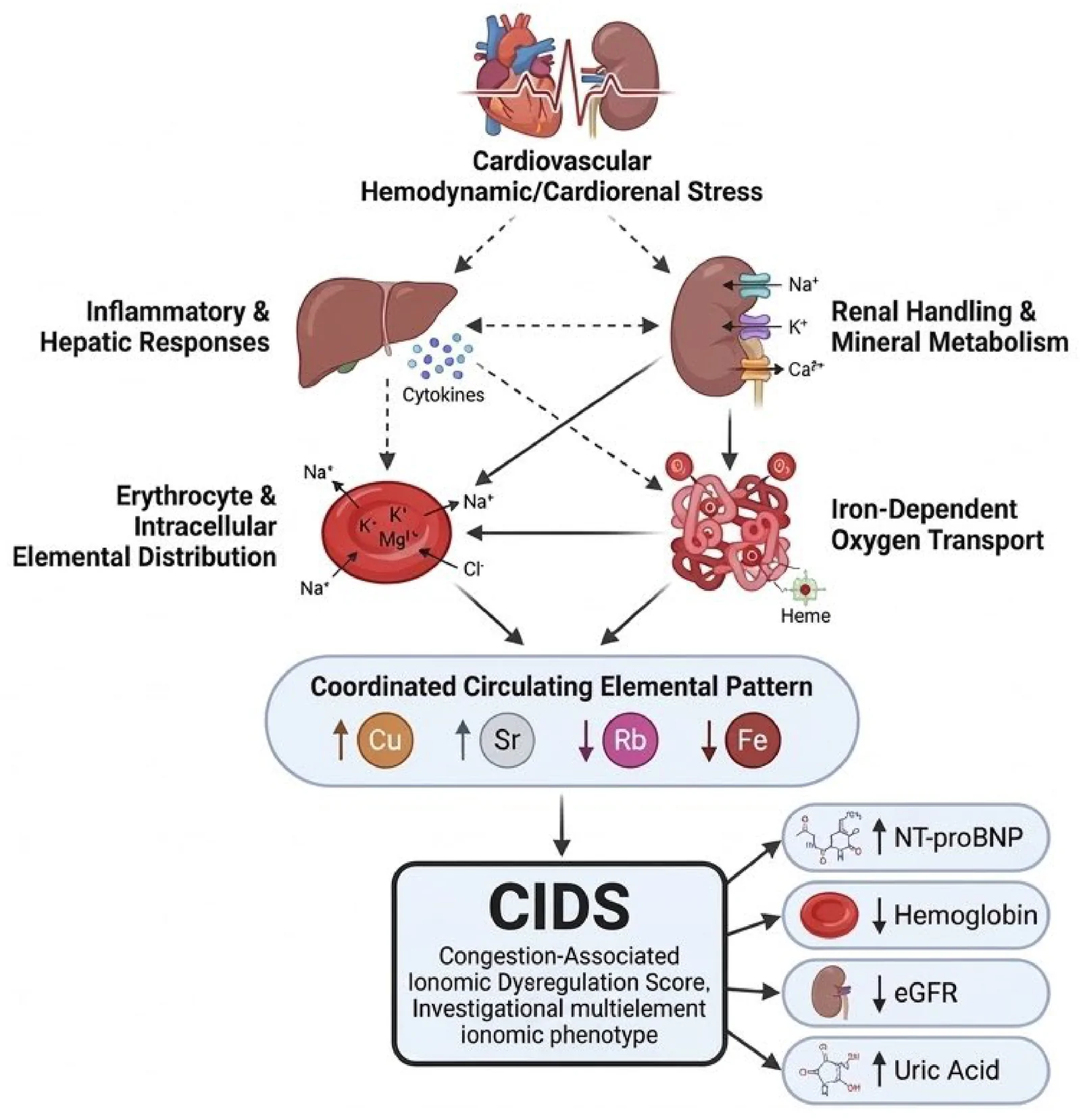
Proposed conceptual framework for the congestion-associated ionomic composite and the congestion-associated ionomic dysregulation score (CIDS). Dashed arrows denote hypothesized biological pathways, whereas solid arrows denote the score construction and observed clinical associations. Abbreviations: Ca^2+^, calcium ion; CIDS, congestion-associated ionomic dysregulation score; Cl^−^, chloride ion; Cu, copper; eGFR, estimated glomerular filtration rate; Fe, iron; K^+^, potassium ion; Mg^2+^, magnesium ion; Na^+^, sodium ion; NT-proBNP, N-terminal pro-B-type natriuretic peptide; Rb, rubidium; Sr, strontium.

The hypothesis-driven conceptual framework for the congestion-associated ionomic composite is summarized in Figure 1.

## 2. Results

### 2.1. Study Population and Data Availability

The updated database contained 192 unique patient-level profiles after consolidation of repeated trace-element measurements by cleaned patient name. Age was available for 190 patients; NT-proBNP for 171; hemoglobin for 188; uric acid for 184; and preoperative creatinine and calculated CKD-EPI 2021 eGFR for 185 patients. The dominant clinical symptoms were easy fatigability and shortness of breath on exertion. NYHA functional status was available for all 192 participants. The recorded distribution was NYHA I in 61 (31.8%), I/II in 35 (18.2%), II in 56 (29.2%), II/III in 21 (10.9%), III in 11 (5.7%), III/IV in 5 (2.6%), and IV in 3 (1.6%) participants. Angiographic vessel-disease count was available for 53 patients, whereas postoperative renal measurements and AKI status were available for 40 patients. Vessel-disease and postoperative renal analyses were considered exploratory. Baseline demographic, comorbidity, laboratory, and angiographic characteristics are summarized in Table 1.

NT-proBNP increased progressively across the ordered NYHA categories (Spearman ρ = 0.882, *p* < 0.001) and (Kruskal–Wallis H = 144.77, *p* < 0.001), supporting a close correspondence between the recorded functional classification and biochemical myocardial stress.

### 2.2. Clinical Burden Across CIDS Quartiles

The 192 patients were divided into four equally sized CIDS quartiles (n = 48 each). Median NT-proBNP increased from 102.1 pg/mL in Q1 to 371.6 pg/mL in Q4. Over the same quartiles, median hemoglobin decreased from 9.30 to 8.65 mmol/L, median preoperative eGFR decreased from 92.2 to 76.8 mL/min/1.73 m^2^, and median uric acid increased from 4.9 to 6.0 mg/dL. The proportion of patients with heart failure increased numerically from 14.9% in Q1 to 29.8% in Q4. Vessel-disease count and postoperative AKI proportions did not show an evident graded increase across quartiles, although these variables were available only in subsets (Table 2).

**Figure 2:**
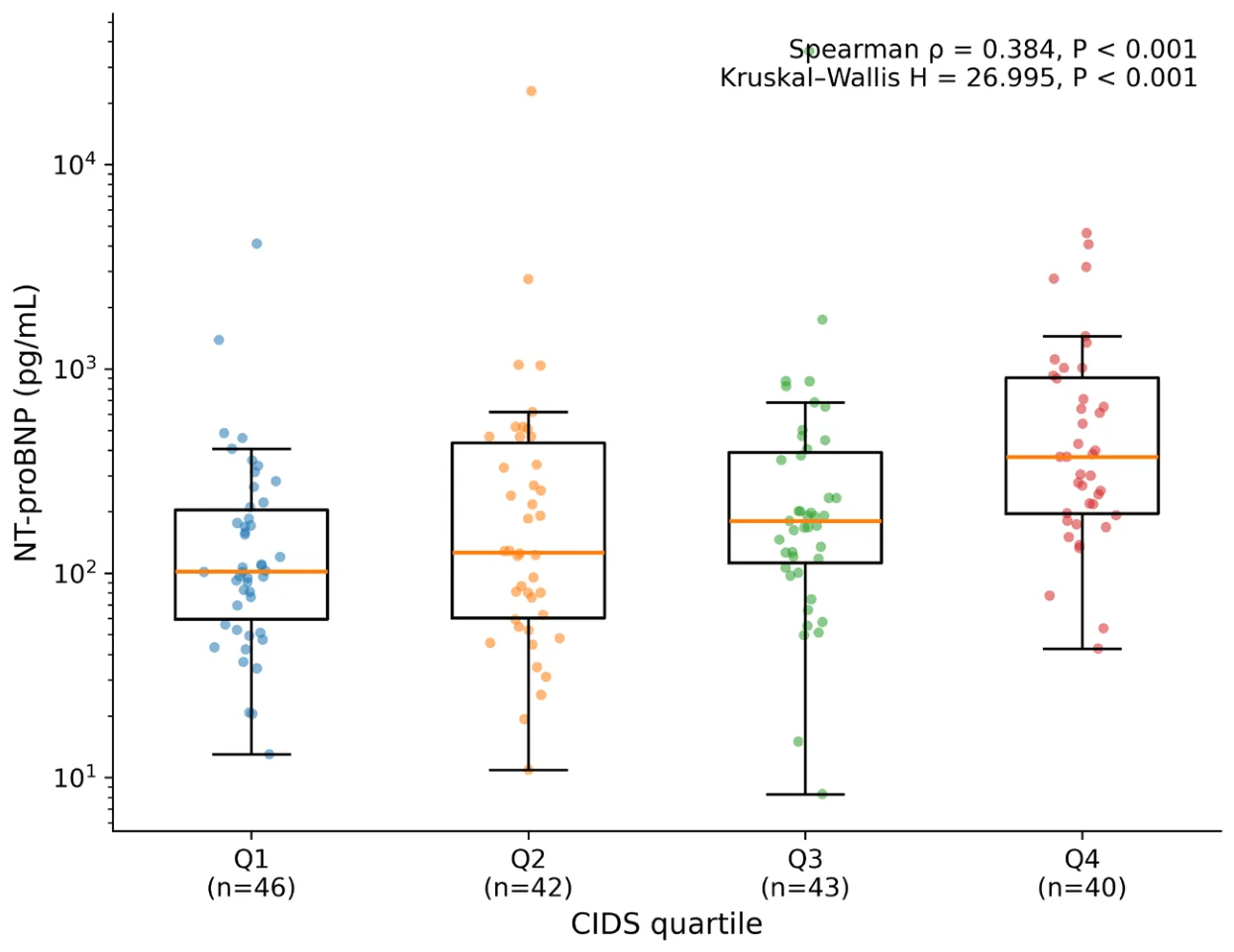
NT-proBNP across quartiles of the congestion-associated ionomic dysregulation score (CIDS). Boxes show the median and interquartile range, whiskers show the conventional 1.5 × interquartile-range limits, and points represent individual patients. Kruskal–Wallis H = 26.995, *p* < 0.001; age- and sex-adjusted trend *p* < 0.001.

NT-proBNP differed across quartiles (Kruskal–Wallis H = 26.995, *p* < 0.001) and increased monotonically with quartile number (Spearman ρ = 0.384, *p* < 0.001). The association also remained significant in an age- and sex-adjusted trend model (β = 0.384 per quartile increase, *p* < 0.001; Figure 2).

### 2.3. Association of CIDS with Clinical Markers

Raw CIDS was positively associated with NT-proBNP (ρ = 0.416, FDR-adjusted *p* < 0.001) and uric acid (ρ = 0.241, FDR-adjusted *p* = 0.002), and inversely associated with hemoglobin (ρ = −0.287, FDR-adjusted *p* < 0.001) and preoperative eGFR (ρ = −0.191, FDR-adjusted *p* = 0.012). The correlation with preoperative creatinine was positive but did not reach statistical significance (ρ = 0.123, *p* = 0.096).

CIDS was also associated with functional limitation. Among 192 participants with available NYHA classification, higher CIDS was associated with higher NYHA functional class (Spearman ρ = 0.370, *p* < 0.001). CIDS distributions differed significantly across the recorded NYHA categories (Kruskal–Wallis H = 27.50, *p* < 0.001), further supporting an association between the multielement ionomic pattern and functional disease burden, although the independence of this relationship from NT-proBNP was examined in subsequent multivariable analyses.

The association between CIDS and functional limitation persisted after multivariable adjustment. In a proportional-odds ordinal logistic regression including 187 participants, each 1-SD higher CIDS was associated with higher odds of being classified in a worse NYHA functional category after adjustment for age, sex, eGFR, and hemoglobin (OR 1.70, 95% CI 1.27–2.28; *p* < 0.001). When log-transformed NT-proBNP was additionally incorporated into the model (n = 185), the CIDS association was markedly attenuated and was no longer significant (OR 1.01, 95% CI 0.71–1.43; *p* = 0.956), whereas NT-proBNP remained strongly associated with higher NYHA class (*p* < 0.001). This attenuation suggests that the relationship between CIDS and functional limitation substantially overlaps with the myocardial-stress phenotype reflected by NT-proBNP (Supplementary Table S4).

**Figure 3:**
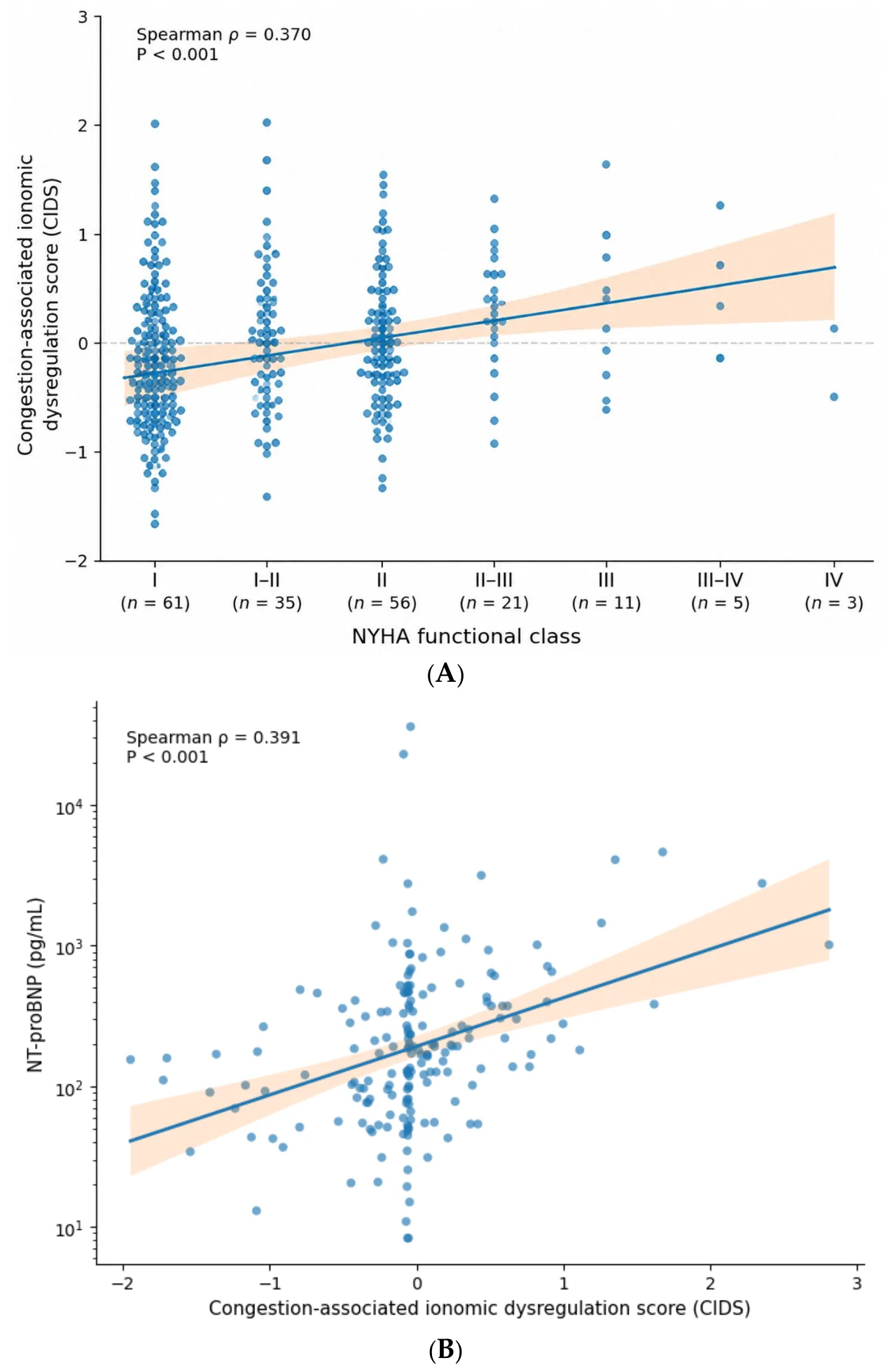
(**A**) Association between the congestion-associated ionomic dysregulation score (CIDS) and New York Heart Association (NYHA) functional status. Points represent patient-level profiles across the seven ordered NYHA categories, including the intermediate categories I/II, II/III, and III/IV retained from the source clinical documentation. The solid line and shaded band represent the fitted trend and 95% confidence interval. Higher CIDS was associated with an increase in NYHA functional class (Spearman ρ = 0.370; *p* < 0.001). (**B**) Association between the congestion-associated ionomic dysregulation score (CIDS) and NT-proBNP. Points represent patient-level profiles; the solid line and shaded band represent the fitted trend and 95% confidence interval. The NT-proBNP axis is logarithmic. Spearman ρ = 0.416; FDR-adjusted *p* < 0.001.

**Figure 4:**
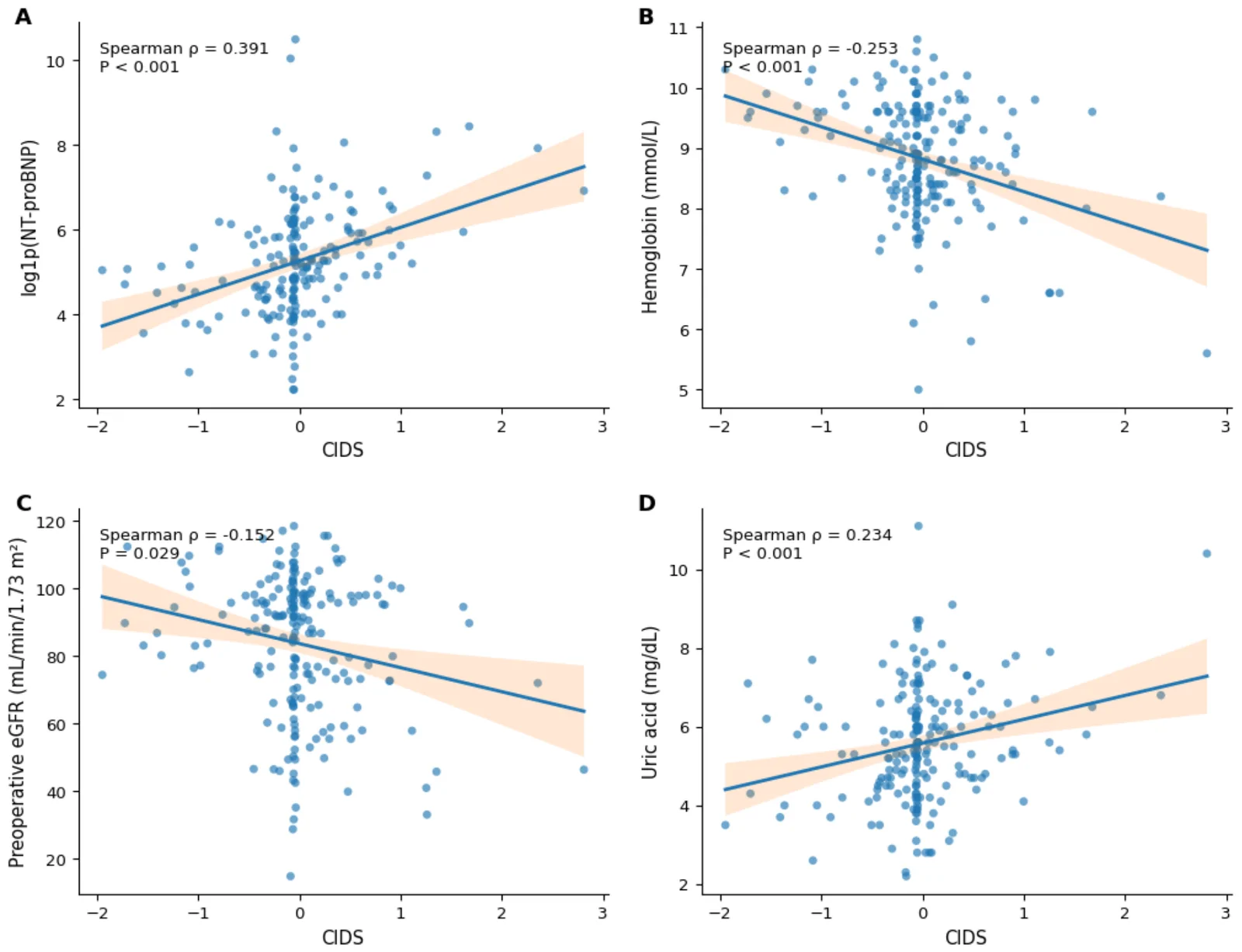
Associations of the congestion-associated ionomic dysregulation score (CIDS) with prespecified clinical markers. NT-proBNP is displayed as log1p(NT-proBNP) (**A**); the other panels show hemoglobin (**B**), preoperative calculated CKD-EPI 2021 eGFR (**C**), and uric acid (**D**). Solid lines and shaded bands represent fitted trends and 95% confidence intervals.

The direct associations between CIDS and NYHA clinical status, and between CIDS and NT-proBNP, are shown in Figure 3A and Figure 3B, respectively. The four relationships with clinical markers are presented in Figure 4.

Results were consistent in the log-transformed sensitivity score. Log-CIDS was associated with NT-proBNP (ρ = 0.439, FDR-adjusted *p* < 0.001), hemoglobin (ρ = −0.294, FDR-adjusted *p* < 0.001), preoperative eGFR (ρ = −0.196, FDR-adjusted *p* = 0.009), preoperative creatinine (ρ = 0.145, FDR-adjusted *p* = 0.049), and uric acid (ρ = 0.316, FDR-adjusted *p* < 0.001; Table 3).

### 2.4. Multivariable Association with NT-proBNP

In the fully adjusted model, each 1-SD increase in raw CIDS was associated with a 0.276-unit increase in log1p(NT-proBNP) (95% CI 0.077–0.475; HC3-robust *p* = 0.007). When CIDS was analyzed in its original units, the corresponding association was β = 0.480 (95% CI 0.134–0.827; HC3-robust *p* = 0.007). Sequential adjustment attenuated but did not abolish the association after inclusion of age, sex, preoperative eGFR, hemoglobin, heart failure, and diabetes. Log-CIDS showed a concordant pattern (Table 4).

In the age- and sex-adjusted model, each 1-unit increase in raw CIDS was associated with a 0.712-unit increase in log1p(NT-proBNP) (95% CI 0.447–0.976; *p* < 0.001), corresponding to an approximately 2.04-fold difference in NT-proBNP + 1. The association remained significant after adjustment for preoperative eGFR and hemoglobin (β = 0.550, 95% CI 0.180–0.920; *p* = 0.004) and after further adjustment for heart failure and diabetes (β = 0.480, 95% CI 0.134–0.827; *p* = 0.007). The adjusted R^2^ increased from 0.213 in Model 1 to 0.382 in Model 3. Log-CIDS showed the same pattern (Table 4).

When expressed per 1-SD higher raw CIDS, the association remained significant in the fully adjusted model (β = 0.276, 95% CI 0.077–0.475; HC3-robust *p* = 0.007). Incremental model comparisons used the same complete-case sample of 170 participants. The clinical reference model explained 36.8% of the variance in log1p(NT-proBNP) (R^2^ = 0.368; adjusted R^2^ = 0.345). Adding standardized CIDS increased R^2^ to 0.407 (ΔR^2^ = 0.039) and adjusted R^2^ to 0.382. The standardized CIDS coefficient was 0.276 (95% CI 0.077–0.475; HC3-robust *p* = 0.007).

Among the individual elements, Sr produced the largest incremental contribution (β = 0.243 per 1 SD; 95% CI 0.054–0.432; *p* = 0.013; ΔR^2^ = 0.034). Cu was not independently associated after adjustment (β = 0.145; *p* = 0.087; ΔR^2^ = 0.012), and Rb (β = 0.078; *p* = 0.324; ΔR^2^ = 0.004) and Fe (β = 0.027; *p* = 0.733; ΔR^2^ < 0.001) added little explanatory information.

The model containing all four standardized elements increased R^2^ to 0.422 (ΔR^2^ = 0.054; adjusted R^2^ = 0.386). The single-score CIDS model had a lower AIC than the four-element model (504.32 vs. 506.05), indicating greater parsimony, but Sr accounted for a substantial part of the incremental association. These comparisons do not establish synergistic biological coordination among the four components (Supplementary Table S1).

After referral group was added to the fully adjusted model, the CIDS association remained significant (β = 0.256 per 1 SD, 95% CI 0.049–0.463; HC3-robust *p* = 0.016). The interaction estimate was imprecise and not significant (β = 0.275, 95% CI −0.117–0.667; *p* = 0.171). Stratified estimates differed in magnitude: group 0 had β = 0.179 (95% CI −0.085–0.443; *p* = 0.187; n = 111), whereas group 1 had β = 0.569 (95% CI 0.112–1.026; *p* = 0.018; n = 59). The pathways also differed in sex distribution (standardized mean difference [SMD] = −0.545), Fe concentration (SMD = 0.353), and heart-failure prevalence (SMD = 0.236). These analyses support retention of the adjusted pooled estimate but do not establish homogeneous effects across pathways (Supplementary Table S8).

Internal validation of the fully adjusted standardized raw-CIDS model using 2000 patient-level bootstrap samples supported the internal stability of the association. The mean bootstrap coefficient was 0.283, with a median of 0.286 and a percentile-based 95% bootstrap confidence interval of 0.099–0.454. The CIDS coefficient remained positive in 99.7% of bootstrap resamples. The apparent model R^2^ was 0.407; mean estimated optimism was 0.066, yielding an optimism-corrected R^2^ of 0.341. Thus, although some reduction in model performance was observed after correcting for optimism, the direction and magnitude of the CIDS association remained stable across repeated resampling (Supplementary Table S2).

The association between CIDS and NT-proBNP was also robust to the exclusion of extreme NT-proBNP observations. In the complete-case sample of 170 participants, the 99th percentile of NT-proBNP was 10,300.7 pg/mL. Two participants with values above this threshold were excluded, leaving 168 participants for sensitivity analysis. In the fully adjusted sensitivity model, the association of raw CIDS with log1p(NT-proBNP) remained significant and was numerically stronger (β = 0.601, 95% CI 0.310–0.892; HC3-robust *p* < 0.001). When CIDS was expressed per 1-SD increment, the corresponding estimate was β = 0.345 (95% CI 0.178–0.512; HC3-robust *p* < 0.001).

These findings indicate that the principal CIDS–NT-proBNP association was not driven by the small number of patients with extreme NT-proBNP concentrations (Supplementary Table S3).

### 2.5. Permutation Analysis of Alternative Four-Element Composites

The observed CIDS increased R^2^ by 0.039. Across all 16 equal-weight sign patterns, the median null ΔR^2^ was 0.015, and the empirical *p* value was 0.176. Across 10,000 randomly weighted composites, the median null ΔR^2^ was 0.011, and the 95th percentile was 0.041; the observed specification did not clearly exceed this distribution (empirical *p* = 0.063). Thus, although CIDS showed an association with NT-proBNP and performed better than the typical alternative weighting, the permutation analyses did not demonstrate that its incremental explanatory value was clearly or uniquely superior to that of alternative four-element composites (Supplementary Table S7).

### 2.6. Single-Element and Ionome-Wide Analyses

At the individual-element level, Cu and Sr showed nominal positive correlations with NT-proBNP (Cu: ρ = 0.158, *p* = 0.038; Sr: ρ = 0.172, *p* = 0.025), but neither remained significant after FDR adjustment across the four CIDS components (both FDR-adjusted *p* = 0.077). Rb and Fe were positively associated with hemoglobin after FDR adjustment (both FDR-adjusted *p* = 0.016), whereas their individual associations with NT-proBNP were weak. Full component–marker correlations are shown in Table 5.

**Figure 5:**
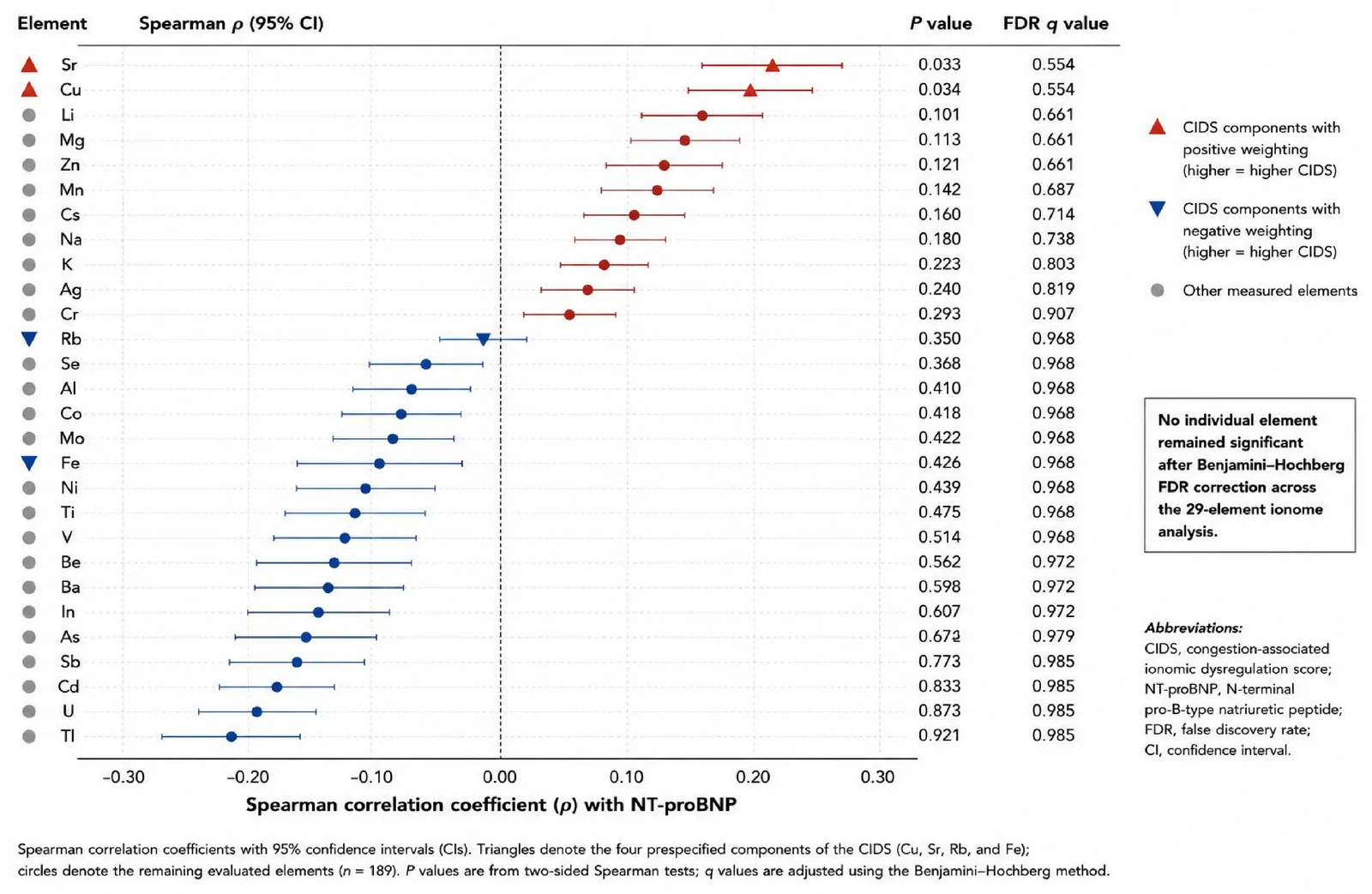
Ionome-wide association profile of circulating elements with NT-proBNP.

In the ionome-wide scan of 29 elements, no individual element remained associated with NT-proBNP after Benjamini–Hochberg correction. The smallest FDR-adjusted *p* values were 0.558 for Sr and Cu. The CIDS composite showed a stronger association than any single element, but the permutation analysis did not establish that its weighting was uniquely superior (Figure 5 and Supplementary Table S7).

Spearman correlations between individual circulating elements and NT-proBNP. Triangles denote the four CIDS components (Cu, Sr, Rb, and Fe), and circles denote the remaining measured elements. The dashed vertical line denotes ρ = 0. No individual element remained significant after Benjamini–Hochberg correction across the ionome-wide analysis.

### 2.7. Exploratory Cardiac Phenotype and Echocardiographic Analyses

Heart failure status was available for 190 participants, of whom 43 had documented heart failure. Raw CIDS was not significantly associated with heart failure in the age-, sex-, and referral-group-adjusted logistic model (OR, 1.61 per 1-unit increase; 95% CI, 0.74–3.51; HC3-robust *p* = 0.228; FDR-adjusted *p* = 0.303). Log-CIDS showed a nominal association with heart failure (OR, 11.91; 95% CI, 1.66–85.26; HC3-robust *p* = 0.014), but this association did not remain significant after FDR correction (FDR-adjusted *p* = 0.055).

Among the available echocardiographic measures, E/e′ was available for 49 participants, RVSP for 102, LVEF for 151, and LAVI for 19. In the age-, sex-, and referral-group-adjusted model, higher raw CIDS was associated with higher E/e′ (β, 1.04 per 1-unit increase in CIDS; 95% CI, 0.19–1.89; HC3-robust *p* = 0.021). However, this association did not remain significant after FDR correction across the four exploratory cardiac phenotype outcomes (FDR-adjusted *p* = 0.085) and was not statistically significant when log-CIDS was used (β, 2.03; HC3-robust *p* = 0.151) (Table 6).

CIDS was not significantly associated with RVSP (β, 1.35; 95% CI, −0.71 to 3.41; HC3-robust *p* = 0.203; FDR-adjusted *p* = 0.271) or LVEF (β, −0.74 percentage points; 95% CI, −3.11 to 1.63; HC3-robust *p* = 0.539; FDR-adjusted *p* = 0.539). LAVI showed no unadjusted association with CIDS (Spearman ρ = 0.055; *p* = 0.822); multivariable analysis was not undertaken because only 19 measurements were available.

### 2.8. Exploratory Angiographic Vessel-Disease Analyses

Among the 53 patients with available angiographic vessel-count data, raw CIDS showed a weak inverse correlation with the number of diseased vessels (Spearman ρ = −0.281, *p* = 0.041). The corresponding association for log-CIDS was similar in magnitude but did not reach nominal statistical significance (ρ = −0.268, *p* = 0.052). Given that angiographic vessel-disease data were available for only 53 participants, along with multiple comparisons and the lack of a consistent adjusted association, these analyses were exploratory and hypothesis-generating and should not be interpreted as evidence of an independent relationship between CIDS and anatomical coronary disease burden. CIDS was also not significantly associated with multivessel disease or three-vessel disease in age- and sex-adjusted logistic models (Supplementary Table S5). The distribution of CIDS by vessel-disease count is shown in Supplementary Figure S1.

### 2.9. Exploratory Perioperative Renal and AKI Analyses

The exploratory postoperative renal analyses were restricted to 40 participants and included only eight AKI events. Given the small sample size and limited number of events, the resulting estimates were extremely imprecise and statistically non-informative; they are reported only in Supplementary Table S6 for transparency and should not be interpreted as evidence for or against an association between CIDS and postoperative AKI.

## 3. Discussion

In this cardiovascular cohort, higher CIDS was associated with NT-proBNP, lower hemoglobin, lower eGFR, and higher uric acid. The adjusted NT-proBNP association remained significant across the primary and sensitivity models. However, Sr accounted for a substantial portion of the incremental association, and permutation analyses did not establish that the observed four-element weighting was uniquely superior to alternative specifications. CIDS should therefore be interpreted as a hypothesis-driven composite summary evaluated in this dataset, not as evidence of confirmed biological coordination among the four elements.

From a metallomic perspective, recent ICP-MS-based studies illustrate the value of evaluating disease-associated multielement patterns rather than interpreting each elemental concentration in isolation; however, such patterns remain context- and matrix-dependent and require independent validation before being considered disease-specific signatures [29].

The most consistent observation was the association between CIDS and NT-proBNP across correlation, quartile-based, and adjusted analyses. No individual element survived the ionome-wide FDR correction, although Sr remained independently associated with the adjusted incremental model. Bootstrap and influence analyses supported internal stability, while the permutation results and referral-stratified estimates showed important specification and population uncertainty. Together, these findings justify further evaluation of CIDS but do not validate it as a biomarker.

CIDS may summarize several elemental changes accompanying cardiac, renal, hematologic, or metabolic burden. The cross-sectional data cannot determine whether these changes share a common mechanism or simply covary with clinical severity. From an ionomic perspective, the rationale for such a composite is to evaluate covariation among multiple elements within a shared analytical framework rather than to interpret each element as an isolated exposure; however, multielement aggregation does not itself establish biological coordination and requires comparison with individual elements and alternative score specifications [4,5].

The association with NYHA functional status provides a complementary clinical context. CIDS remained associated with worse functional status after adjustment for age, sex, renal function, and hemoglobin; however, this association was abolished after inclusion of NT-proBNP. Together with the very strong relationship between NT-proBNP and NYHA class observed in this cohort, this finding suggests that the CIDS–NYHA association largely tracks the same myocardial-stress phenotype captured by NT-proBNP rather than representing an independent dimension of functional impairment.

The association between CIDS and NT-proBNP is compatible with several, non-exclusive explanations. Hemodynamic burden could influence renal filtration, inflammatory or hepatic responses, erythropoiesis, and elemental distribution, but diet, medications, comorbidities, or other unmeasured factors could also account for the observed pattern. The modest correlations with renal function, hemoglobin, and uric acid do not identify specific physiological axes. CIDS should not be interpreted as causing NT-proBNP elevation or as a direct measure of filling pressure.

A composite index and a single-element biomarker answer different questions. CIDS summarized four directional components in one variable and retained most of the explanatory information in the less parsimonious four-element model. However, the permutation analyses did not demonstrate that the incremental explanatory value of the prespecified CIDS was clearly superior to that of alternative four-element composites. Accordingly, the present findings support CIDS as an exploratory multielement phenotype associated with NT-proBNP, but not as a validated biomarker, predictive signature, or uniquely informative ionomic score. Independent validation in an external cohort, preferably with prospectively defined score construction and assessment of predictive performance, would be required before such interpretations could be justified. The lack of ionome-wide significance for individual elements also argues against presenting Cu, Sr, Rb, or Fe alone as a diagnostic marker. Independent validation is required for both the composite specification and its clinical interpretation.

The association with NYHA functional status provides an additional clinical dimension to the CIDS phenotype. Higher CIDS was associated with progressively greater functional limitation, suggesting that the ionomic pattern identified here is not restricted to variation in a circulating biomarker but also tracks patient-level symptomatic burden. This finding is particularly relevant because NYHA classification and NT-proBNP capture related but non-identical aspects of cardiovascular disease: NT-proBNP primarily reflects myocardial wall stress, whereas NYHA class reflects the clinical consequences of cardiovascular limitation during daily activity. The concordant associations of CIDS with both measures therefore support its interpretation as a systemic phenotype accompanying greater cardiovascular burden. Nevertheless, NYHA classification is subjective and was retrospectively abstracted, and the present findings should be considered exploratory.

The incremental analysis showed that CIDS increased the model’s R^2^ by 0.039 relative to the clinical reference model and yielded a lower AIC than the model containing four separate elemental terms. Sr provided nearly as much incremental R^2^ as CIDS, whereas Cu, Rb, and Fe contributed little when added separately. Accordingly, the present value of CIDS lies in its parsimonious representation of a prespecified four-element pattern associated with NT-proBNP, rather than in demonstrated statistical superiority over its individual constituents or alternative four-element composites. These data should not be interpreted as establishing CIDS as a predictive signature or clinically validated biomarker.

The positive Cu component was directionally consistent with previous heart-failure studies [10,11,12,14], but Cu was not independently significant in the adjusted incremental model. Because digestion-based ICP-MS measured total Cu rather than ceruloplasmin or Cu species, the present data cannot attribute the association to Cu excess, a particular oxidation state, inflammation, or hepatic congestion. Those explanations remain hypotheses requiring direct measurements.

Sr provided the strongest individual incremental association with NT-proBNP. Renal elimination, dietary or environmental exposure, calcium–phosphate metabolism, and bone turnover may influence circulating Sr [23,24,25], but the observed association does not identify which process was responsible. Urinary excretion, dietary assessment, and mineral metabolism measurements are needed to distinguish among these explanations.

Rb and Fe were associated with hemoglobin in unadjusted correlation analyses, but neither contributed materially to the adjusted NT-proBNP model. Their relationships with erythrocyte mass, nutrition, cellular distribution, or iron metabolism remain exploratory. Total Fe measured by ICP-MS is not equivalent to ferritin, transferrin saturation, functional iron deficiency, or a specific Fe oxidation state and should not be interpreted as an indication for iron treatment.

Exploratory cardiac phenotype analyses did not provide FDR-corrected evidence of an association between CIDS and cardiac hemodynamic measures. Higher raw CIDS was associated with higher E/e′ after adjustment for age, sex, and referral group. However, this association did not remain significant after FDR correction and was not reproduced with log-CIDS. Log-CIDS showed a nominal association with heart failure, but this result also did not remain significant after FDR correction and had a wide confidence interval. CIDS was not associated with RVSP or LVEF, and LAVI data were sparse. Overall, these findings are hypothesis-generating and do not establish CIDS as a direct marker of filling pressure or congestion [6,30].

Bootstrap and influence analyses supported internal stability of the adjusted association, but they do not provide external validation. Referral-stratified estimates differed in magnitude, and the permutation analysis did not show clear superiority of the selected weighting. These results narrow the conclusion to an internally observed association that requires replication in an independent cohort.

The angiographic analyses were exploratory and did not establish an association with anatomical coronary burden. The postoperative renal analysis, based on 40 participants and eight AKI events, was too underpowered and imprecise to provide interpretable evidence and is retained only in the Supplementary Materials for transparency.

The analysis used raw and log-transformed formulations, calculated CKD-EPI eGFR, performed HC3-robust inference, conducted influence analysis, and performed bootstrap resampling. The additional complete-case, permutation, case-mix, and referral-stratified analyses clarify the stability and limits of the main association. These checks do not overcome the score’s exploratory construction or the absence of external validation.

An additional barrier to clinical translation is the cohort-specific construction of CIDS. Because each component was standardized using the mean and standard deviation of the present analytical cohort, a given CIDS value is relative to this study population and cannot be directly transferred to another cohort. No externally derived reference distribution, clinically validated cutoff, or decision threshold currently exists. CIDS should therefore be regarded as a research phenotype rather than a clinical diagnostic, prognostic, or treatment-guiding score. Translation into clinical practice would require external standardization, independent validation, assessment of assay reproducibility across laboratories, and prospective evaluation of clinically meaningful thresholds.

### Limitations

This was a single-center, observational, cross-sectional analysis, so temporal order and causality cannot be determined: higher CIDS may precede congestion-related organ dysfunction, follow it, or arise concurrently from shared or unmeasured causes. The score was constructed and evaluated in the same cohort without documented preregistration, and the permutation analyses did not establish unique superiority over alternative weightings. The two referral pathways differed in case mix and produced different point estimates in stratified analyses; the non-significant interaction does not prove homogeneity. Dietary and water exposure, supplements, transfusion history, detailed medication exposure including diuretic use, hepatic function, ceruloplasmin, complete iron-status measures, inflammatory markers including C-reactive protein (CRP), and standardized sampling time were unavailable or incomplete. Residual confounding by these factors may therefore partly account for the observed relationships between CIDS, its individual elemental components, and clinical measures. Echocardiographic, angiographic, and postoperative renal data were sparse, and the postoperative AKI analysis was statistically non-informative. No longitudinal outcomes were available. The study therefore does not establish prognostic utility, diagnostic thresholds, causal mechanisms, or incremental clinical value over established biomarkers. Cohort-specific z-standardization also prevents direct transport of absolute CIDS values to another population.

Complete-case analysis without imputation may also have introduced selection bias if the availability of laboratory, echocardiographic, angiographic, or postoperative variables was related to disease severity, referral pathway, or other patient characteristics. This concern is particularly relevant to exploratory analyses based on substantially smaller subsets, including echocardiographic measures and postoperative renal outcomes. These subset analyses may therefore not be representative of the full 192-patient cohort and should be interpreted as hypothesis-generating only.

## 4. Materials and Methods

### 4.1. Study Design and Participants

This retrospective observational cohort study included a cross-sectional primary analysis and was conducted at Poznan University of Medical Sciences in Poznan, Poland, between 2025 and 2026. The source population comprised patients referred for inpatient cardiovascular evaluation or treatment from the Department of Hypertensiology, Angiology and Internal Medicine or the Department of Cardiac Surgery and Transplantology. Patients were classified according to referral pathway rather than into mutually exclusive diagnostic groups. The predominant clinical symptoms were exertional dyspnea and fatigue.

After linking the clinical and trace-element datasets and performing patient-level aggregation, 192 patients were included. Of these, 118 (61.5%) were referred from the Department of Hypertensiology, Angiology and Internal Medicine, and 74 (38.5%) from the Department of Cardiac Surgery and Transplantology. The cohort included 124 men (64.6%) and 68 women (35.4%); age was available for 190 participants, with a median age of 68 years (interquartile range, 62–73 years). Ischemic heart disease and heart failure were documented in 164 (86.3%) and 43 participants (22.6%), respectively, with both diagnoses present in 36 (18.9%). Among participants referred from cardiac surgery, 50 of 74 (67.6%) underwent CABG during the index treatment episode.

Admissions were elective and related to coronary angiography, possible percutaneous coronary intervention, assessment of a coronary revascularization strategy, planned CABG, or preoperative cardiovascular evaluation. A smaller proportion involved valvular or aortic intervention, acute coronary syndrome, procedural complications, or worsening cardiovascular symptoms. Presenting symptoms commonly included angina or chest discomfort, dyspnea, fatigue, and reduced exercise tolerance; signs suggestive of congestion were documented in a subset of patients. Clinical and laboratory data were retrospectively abstracted from electronic medical records and discharge letters, including admission characteristics, diagnoses and comorbidities, medication use, laboratory and echocardiographic findings, angiographic data, and operative or postoperative information where applicable.

The two referral pathways were analyzed as a single cardiovascular cohort because they represented overlapping clinical populations rather than mutually exclusive disease groups. Both pathways included patients evaluated for ischemic heart disease, coronary revascularization, heart failure, and related cardiovascular conditions. Because the primary objective concerned the association between a continuous patient-level ionomic phenotype and NT-proBNP rather than differences between departments, the referral source was treated as a potential source of heterogeneity and examined in sensitivity and interaction analyses.

### 4.2. Clinical Variables and Outcome Definitions

Recorded clinical variables included age, sex, body mass index, heart failure status, diabetes mellitus, hemoglobin, uric acid, NT-proBNP, E/e′ ratio, left atrial volume index (LAVI), estimated right ventricular systolic pressure (RVSP), left ventricular ejection fraction (LVEF), preoperative creatinine, and angiographic vessel-disease count. Heart failure and diabetes diagnoses were abstracted from the treating physicians’ documented clinical diagnoses and the applicable clinical criteria [6].

Routine laboratory measurements were obtained on admission. Blood for trace-element analysis was collected after overnight fasting and processed independently of the clinical dataset. Hematological parameters were measured using a routine automated hematology analyzer (Sysmex Euro GmbH, Norderstedt, Germany).

NT-proBNP concentration was the primary outcome, followed by secondary clinical markers: hemoglobin, preoperative estimated glomerular filtration rate (eGFR), preoperative creatinine, and uric acid. Exploratory cardiac phenotype analyses included heart failure status and available echocardiographic parameters: E/e′ ratio, LAVI, RVSP, and LVEF. When LVEF was reported as a range, the midpoint of that range was used for continuous-variable analyses. Angiographic vessel-disease count and perioperative renal outcomes were considered exploratory because these variables were available only in subsets of the cohort.

NYHA functional class was abstracted from the clinical documentation as recorded by the treating physician. In addition to the conventional NYHA classes I–IV, the source records contained intermediate categories I/II, II/III, and III/IV. These categories were retained as ordered intermediate levels for the exploratory analysis (coded 1.0, 1.5, 2.0, 2.5, 3.0, 3.5, and 4.0, respectively) rather than being reassigned to conventional NYHA classes.

### 4.3. Biological Sample Collection and Trace-Element Quantification

Venous blood was collected after overnight fasting. Serum was separated by centrifugation at 4000× *g* for 5 min at room temperature and stored at −80 °C until trace-element analysis.

To prepare samples for analysis, 500 µL aliquots of serum were transferred into disposable DigiTube vials (SCP Science Ltd., Baie-D’Urfé, QC, Canada), followed by the addition of 2 mL of 65% nitric acid and 1 mL of 30% hydrogen peroxide. Wet digestion was performed at 150 °C for 4 h using an AccuBlock Digital heating block system (Labnet, Edison, NJ, USA). Following digestion and subsequent cooling, the mixtures were diluted 80-fold with ultrapure water. Procedural blanks and certified reference materials were processed in parallel using the identical digestion protocol. The resulting solutions were stored in polypropylene tubes at 4 °C prior to analysis.

Trace-element concentrations were quantified on an Agilent 7700× quadrupole ICP-MS system (Agilent Technologies, Tokyo, Japan) equipped with an Octopole Reaction System (ORS). Polyatomic interferences were minimized by operating the ORS in helium collision mode. Instrumental parameters were kept constant during all analytical runs: RF power of 1550 W, with plasma, nebulizer, and auxiliary gas flows maintained at 15 L min^−1^, 0.98 L min^−1^, and 0.9 L min^−1^, respectively. Physical transport effects and instrument signal drift were corrected using rhodium (10 µg L^−1^) and terbium (10 µg L^−1^) as internal standards. Calibration was performed externally utilizing matrix-matched multi-element standards (Calibration Std 3, PerkinElmer, Waltham, MA, USA) prepared in 3.25% (*v*/*v*) HNO_3_. The complete analytical panel comprised Li, Be, Na, Mg, Al, K, Ti, V, Cr, Mn, Fe, Co, Ni, Cu, Zn, As, Se, Rb, Sr, Mo, Ag, Cd, In, Sb, Cs, Ba, Tl, Pb, and U. The four CIDS components were Cu, Sr, Rb, and Fe; the remaining elements were included in the exploratory ionome-wide analysis. The digestion-based ICP-MS procedure quantified total elemental concentrations. It did not preserve or distinguish oxidation states, ionic forms, molecular species, or protein-bound fractions. Accordingly, elemental symbols throughout the manuscript (Cu, Sr, Rb, and Fe) refer to total measured elemental concentrations rather than specific oxidation states or ionic species; physiologically relevant forms may include Cu^+^/Cu^2+^, Sr^2+^, Rb^+^, and Fe^2+^/Fe^3+^. Total Cu and total Fe measured by this method should therefore not be interpreted as equivalent to ceruloplasmin, ferritin, transferrin saturation, or clinical iron-deficiency measures.

Method validation and quality control relied on Seronorm Trace Elements Serum L1 and L2 certified reference materials (Billingstad, Norway) to evaluate analytical trueness and establish traceability. Six-point calibration curves showed excellent linearity (R^2^ > 0.999), spanning concentration ranges of 1–500 µg L^−1^ for Mg, Fe, and K, and 0.05–100 µg L^−1^ for the other analytes. Limits of detection (LODs), derived from the calibration parameters, ranged from 0.008 µg L^−1^ for Cu to 0.5 µg L^−1^ for Mg. Analytical precision was determined from triplicate analyses of the reference materials, yielding coefficients of variation (CV) of 1.5% for Fe and 5% for Zn. Trueness, expressed as recovery of certified concentrations, ranged from 88% for Cr to 112% for Al. Student’s *t*-test confirmed that the measured values (mean ± SD) agreed with the certified concentrations (mean ± standard uncertainty) within statistical limits.

### 4.4. Construction of the Congestion-Associated Ionomic Dysregulation Score

CIDS was constructed as a hypothesis-driven pattern of relatively higher copper (Cu) and strontium (Sr) and lower rubidium (Rb) and iron (Fe). For each element, patient-level concentrations were standardized within the analytic cohort to a mean of 0 and a standard deviation of 1. The primary score was calculated from raw-concentration z scores as follows:

CIDS = (ZCu + ZSr)/2 − (ZRb + ZFe)/2

Equal weighting was used to avoid optimizing component weights with respect to NT-proBNP in this cohort. Because the score was constructed and evaluated in the same dataset and no dated preregistration or institutional analysis plan was available for verification, CIDS is described as a hypothesis-driven exploratory composite rather than a formally prespecified or externally validated score.

Accordingly, higher CIDS values indicate a stronger Cu-high/Sr-high/Rb-low/Fe-low ionomic pattern. A log-transformed CIDS (log-CIDS) was calculated using the same equation after applying the natural logarithm to each element concentration, followed by z-score standardization. Both scores were analyzed in parallel, with the raw CIDS serving as the primary analysis and the log-CIDS as a sensitivity analysis.

### 4.5. Echocardiographic Variables

Comprehensive transthoracic echocardiography was performed by experienced cardiologists using commercially available ultrasound systems, in accordance with the recommendations of the European Association of Cardiovascular Imaging. Standard two-dimensional, Doppler, and tissue Doppler imaging were acquired during routine clinical evaluation. Left ventricular ejection fraction (LVEF) was determined using the biplane Simpson method whenever feasible. Left atrial volume index (LAVI), transmitral early filling velocity (E), early diastolic mitral annular velocity (e′), the E/e′ ratio, and estimated right ventricular systolic pressure (RVSP) were assessed using standard guideline-based methodology [30].

### 4.6. Renal and Angiographic Variables

Preoperative eGFR was calculated from preoperative serum creatinine using the 2021 race-free CKD-EPI creatinine equation, incorporating age and sex [31]. The preoperative creatinine value recorded in the study database was used as the baseline measurement. Postoperative peak creatinine was defined as the highest serum creatinine concentration recorded within 7 days after surgery. Postoperative eGFR was calculated from the postoperative peak creatinine using the same 2021 CKD-EPI creatinine equation; therefore, this value represented the lowest calculated eGFR during the postoperative 7-day period. Changes in creatinine and eGFR were calculated as postoperative minus preoperative values.

Postoperative acute kidney injury (AKI) and AKI stage were determined according to the creatinine-based kidney disease: Improving Global Outcomes (KDIGO) criteria [32], using preoperative serum creatinine as the baseline value and postoperative measurements obtained within 7 days after surgery. AKI was defined as an increase in serum creatinine of at least 0.3 mg/dL or at least 1.5 times the baseline value. Stage 1 AKI was defined as an increase to 1.5–1.9 times baseline or an absolute increase of at least 0.3 mg/dL; stage 2 as an increase to 2.0–2.9 times baseline; and stage 3 as an increase to at least 3.0 times baseline or a postoperative serum creatinine concentration of at least 4.0 mg/dL. Urine-output criteria were not applied because sufficiently detailed and standardized urine-output data were not available.

Angiographic coronary disease burden was evaluated using the vessel-disease count recorded in the study database, ranging from 0 to 3. This variable represented the number of major epicardial coronary territories classified as diseased on clinical coronary angiography. The vessel count was analyzed as recorded and was not recalculated from the individual percentage-stenosis variables in the present statistical analysis. Multivessel disease was defined as involvement of at least two vessels, whereas three-vessel disease was analyzed separately. Angiographic analyses were considered exploratory because vessel-count data were available only in a subset of the cohort.

### 4.7. Statistical Analysis

Statistical analyses were performed using R version 4.6.0 (R Foundation for Statistical Computing, Vienna, Austria). HC3 heteroskedasticity-consistent covariance matrices were calculated with sandwich::vcovHC (type = “HC3”), and coefficient tests were obtained with lmtest::coeftest. Nonparametric patient-level bootstrap resampling was implemented with base R sampling functions using 2000 resamples and random seed 20260915. Statistical significance was defined as a two-sided *p* < 0.05 unless otherwise specified. Continuous variables were summarized as mean ± standard deviation or median (interquartile range), whereas categorical variables were summarized as counts and percentages. Spearman’s rank correlation was used to evaluate associations between CIDS and clinical variables. Benjamini–Hochberg correction was applied within the analysis families defined below.

Because participants originated from two referral pathways, we compared available case-mix variables between pathways using standardized mean differences. The fully adjusted NT-proBNP model was then repeated with referral group as a covariate and with a CIDS × referral-group interaction. Fully stratified models with the same covariate structure were reported alongside the pooled analysis. Interaction-test non-significance was not treated as proof of homogeneity because the group-specific estimates were imprecise.

As NT-proBNP showed a markedly right-skewed distribution, an additional influence sensitivity analysis was performed to assess whether the primary association was disproportionately driven by extreme NT-proBNP observations. The 99th percentile of NT-proBNP was determined within the complete-case sample used for the fully adjusted model, and the primary regression was repeated after excluding observations above this threshold. The same covariate structure and HC3 robust standard errors were retained.

To assess explanatory information beyond conventional clinical variables, a clinical reference model included age, sex, calculated eGFR, hemoglobin, heart failure, and diabetes. CIDS, Cu, Sr, Rb, and Fe were added separately after standardization to 1-SD increments. A further model included the four elements simultaneously. R^2^, adjusted R^2^, ΔR^2^, AIC, and HC3-robust coefficient inference were compared. Every nested model was fitted to the identical complete-case sample defined by the outcome, all clinical covariates, CIDS, and the four component elements; no model-specific case deletion was permitted. Values that differ by 0.001 between displays reflect rounding only unless otherwise stated.

The association between CIDS and NYHA functional status was evaluated as an exploratory ordinal analysis using Spearman’s rank correlation across the seven recorded ordered NYHA categories. Differences in CIDS across these categories were assessed using the Kruskal–Wallis test. The association between NT-proBNP and NYHA class was evaluated using the same ordinal framework. A multivariable ordinal logistic regression was additionally used to assess the association between standardized CIDS and higher NYHA functional class after adjustment for clinical covariates.

Two permutation analyses assessed whether the observed CIDS improvement could be replicated using alternative four-element composites. First, all 16 equal-weight sign patterns for Cu, Sr, Rb, and Fe were evaluated. Second, 10,000 normally distributed four-element weight vectors were normalized to the Euclidean length of the observed CIDS weight vector and applied to the same standardized elements. Each candidate’s score was added to the identical complete-case clinical reference model. Empirical *p* values were calculated as (1 + number of null ΔR^2^ values at least as large as the observed ΔR^2^)/(1 + number of null iterations). The random seed was 20260915.

*p* values from correlation analyses were adjusted using the Benjamini–Hochberg false discovery rate (FDR) procedure. Multivariable linear regression models were fitted with log-transformed NT-proBNP as the dependent variable using sequential adjustment for predefined clinical covariates. Robust HC3 standard errors were applied for the primary regression analyses. Exploratory analyses included heart failure status and available echocardiographic measures, vessel-disease burden and perioperative renal outcomes. All statistical tests were two-sided, and *p* < 0.05 was considered statistically significant.

For effect-size comparability, raw CIDS and log-CIDS were additionally standardized to their respective standard deviations within the 192-patient analytical cohort, and the sequential NT-proBNP regression models were repeated with the standardized scores. The fully adjusted standardized raw-CIDS model was subjected to internal validation using 2000 nonparametric patient-level bootstrap resamples with replacement. The bootstrap distribution of the CIDS regression coefficient was used to derive percentile-based 95% confidence intervals and to quantify directional stability as the proportion of bootstrap estimates greater than zero. Model optimism was assessed by comparing R^2^ obtained in each bootstrap sample with the performance of the corresponding bootstrap-fitted model when applied to the original complete-case analytical sample. Mean optimism was subtracted from the apparent R^2^ to obtain the optimism-corrected R^2^.

Analyses were performed using available-case data for descriptive and correlation analyses. Multivariable regression analyses used complete cases for all variables included in the respective model; no missing-value imputation was performed. Sample size is therefore reported separately for each analysis.

### 4.8. Ethical Approval

The study was conducted in accordance with the Declaration of Helsinki and was approved by the Poznan University of Medical Sciences Ethics Committee (protocol code 33/25, dated 9 January 2025).

## 5. Conclusions

In this cardiovascular cohort, higher CIDS was associated with NT-proBNP and several measures of cardiorenal–hematologic burden. The NT-proBNP association remained significant after multivariable adjustment, and the four-element score provided a parsimonious summary of the measured pattern. However, Sr accounted for a substantial portion of the incremental association; permutation analyses did not establish unique superiority of the selected weighting, and referral-stratified estimates differed in magnitude. The group-specific coefficients were β = 0.179 and β = 0.569, with an imprecise interaction test (*p* = 0.171); the pooled estimate may therefore not apply uniformly across referral pathways.

These findings come from a single-center, cross-sectional study in which CIDS was constructed and evaluated without external validation. Because the score relies on cohort-specific z-standardization and has no externally validated reference values or clinical cutoffs, CIDS should currently be considered a research phenotype and is not directly applicable to clinical decision-making. These findings cannot establish whether CIDS elevation precedes or follows organ dysfunction, confirm biological coordination among the components, or support the use of CIDS as a diagnostic or prognostic biomarker. Independent longitudinal validation with direct measurements of hemodynamic, inflammatory, renal, exposure, and clinical iron status is required.

## Figures and Tables

**Table 1:** Baseline characteristics of the study population.

Characteristic	Available n	n (%) or Median [IQR]	Mean ± SD
Demographics			
Age, years	*190*	68.00 [62.00, 73.00]	66.46 ± 9.49
Sex, male	*192*	124/192 (64.6%)	—
Sex, female	*192*	68/192 (35.4%)	—
Body mass index, kg/m^2^	*107*	27.40 [25.00, 30.36]	28.11 ± 4.62
Comorbidities			
Hypertension	*190*	133/190 (70.0%)	—
Dyslipidemia	*190*	147/190 (77.4%)	—
Chronic kidney disease	*190*	15/190 (7.9%)	—
Chronic obstructive pulmonary disease	*190*	8/190 (4.2%)	—
Peripheral artery disease	*190*	13/190 (6.8%)	—
Ischemic heart disease	*190*	164/190 (86.3%)	—
Atrial fibrillation	*190*	30/190 (15.8%)	—
Heart failure	*190*	43/190 (22.6%)	—
Diabetes mellitus	*190*	68/190 (35.8%)	—
Prior myocardial infarction	*190*	24/190 (12.6%)	—
NYHA functional status			
NYHA I	*192*	61/192 (31.8%)	—
NYHA I/II		35/192 (18.2%)	—
NYHA II		56/192 (29.2%)	—
NYHA II/III		21/192 (10.9%)	
NYHA III		11/192 (5.7%)	
NYHA III/IV		5/192 (2.6%)	
NYHA IV		3/192 (1.6%)	—
Laboratory findings			
White blood cell count, ×10^9^/L	*189*	7.27 [5.80, 8.80]	7.46 ± 2.29
Neutrophil count, ×10^9^/L	*189*	4.50 [3.48, 5.84]	4.82 ± 2.02
Lymphocyte count, ×10^9^/L	*189*	1.65 [1.36, 2.09]	1.80 ± 0.74
Hemoglobin, mmol/L	*188*	8.85 [8.20, 9.60]	8.77 ± 1.01
Platelet count, ×10^9^/L	*189*	240.00 [203.00, 280.00]	250.59 ± 79.65
Alanine aminotransferase, U/L	*187*	26.00 [19.00, 35.50]	31.77 ± 24.15
Glucose, mg/dL	*187*	100.00 [91.00, 116.00]	107.57 ± 26.40
Total cholesterol, mg/dL	*182*	150.50 [127.00, 180.75]	160.64 ± 49.02
LDL cholesterol, mg/dL	*185*	75.00 [55.00, 109.00]	86.20 ± 42.11
HDL cholesterol, mg/dL	*186*	48.00 [40.00, 60.00]	50.11 ± 13.32
Triglycerides, mg/dL	*186*	100.00 [76.00, 130.00]	114.05 ± 57.46
Sodium, mmol/L	*189*	140.00 [138.00, 141.00]	139.68 ± 2.52
Potassium, mmol/L	*189*	4.33 [4.12, 4.58]	4.38 ± 0.43
Uric acid, mg/dL	*184*	5.50 [4.50, 6.50]	5.56 ± 1.50
HbA1c, %	*56*	6.40 [5.90, 7.15]	6.59 ± 0.99
Serum iron, µg/dL	*62*	98.00 [60.50, 117.25]	94.52 ± 38.06
Ferritin, ng/mL	*50*	175.00 [48.00, 262.25]	189.88 ± 155.99
Transferrin saturation, %	*58*	31.00 [21.50, 41.75]	31.86 ± 13.20
Preoperative creatinine, mg/dL	*185*	0.89 [0.74, 1.06]	0.95 ± 0.35
Preoperative eGFR (CKD-EPI 2021), mL/min/1.73 m^2^	*185*	88.10 [70.10, 98.00]	82.81 ± 20.77
NT-proBNP, pg/mL	*171*	180.30 [84.60, 405.65]	737.26 ± 3289.39
Angiographic data			
Angiographic vessel-disease count	*53*	3.00 [2.00, 3.00]	—
1-vessel disease		8/53 (15.1%)	—
2-vessel disease		12/53 (22.6%)	—
3-vessel disease		33/53 (62.3%)	—

Note: Data are presented as n/N (%) for categorical variables and median [interquartile range] and mean ± standard deviation for continuous variables. BMI (107/192), ferritin (50/192), and transferrin saturation (58/192) had substantial missingness; summaries for these variables may not represent the full cohort and should be interpreted cautiously. Abbreviations: CKD-EPI, Chronic Kidney Disease Epidemiology Collaboration; eGFR, estimated glomerular filtration rate; HbA1c, glycated hemoglobin; HDL, high-density lipoprotein; IQR, interquartile range; LDL, low-density lipoprotein; NT-proBNP, N-terminal pro-B-type natriuretic peptide; SD, standard deviation.

**Table 2:** Clinical burden across quartiles of the congestion-associated ionomic dysregulation score.

CIDS Quartile	n	NT-proBNP, Median [IQR]	Hb, Median	Preoperative eGFR, Median	Uric acid, Median	Heart Failure, n/N (%)	Vessel Count, Median	AKI, n/N (%)
Q1 (lowest)	48	102.1 [59.5, 204.2]	9.30	92.2	4.9	7/47 (14.9%)	3	2/10 (20.0%)
Q2	48	126.6 [55.9, 434.1]	8.90	87.7	5.5	8/48 (16.7%)	3	1/5 (20.0%)
Q3	48	180.3 [112.2, 391.0]	8.60	88.8	5.6	14/48 (29.2%)	3	2/9 (22.2%)
Q4 (highest)	48	371.6 [195.4, 905.9]	8.65	76.8	6.0	14/47 (29.8%)	3	3/16 (18.8%)

Note: AKI data were available for 40 patients, and angiographic vessel disease count was available for 53 patients (surgical cohort). Abbreviations: AKI, acute kidney injury; CIDS, congestion-associated ionomic dysregulation score; eGFR, estimated glomerular filtration rate; IQR, interquartile range.

**Table 3:** Correlations of raw and log-transformed CIDS with clinical markers.

Score	Clinical Marker	n	Spearman ρ	*p* Value	FDR-Adjusted *p*
CIDS-RAW	NT-proBNP	*171*	0.416	<0.001	<0.001
CIDS-RAW	Hemoglobin	*188*	−0.287	<0.001	<0.001
CIDS-RAW	Preoperative eGFR	*185*	−0.191	0.009	0.012
CIDS-RAW	Preoperative creatinine	*185*	0.123	0.096	0.096
CIDS-RAW	Uric acid	*184*	0.241	<0.001	0.002
CIDS-LOG	NT-proBNP	*171*	0.439	<0.001	<0.001
CIDS-LOG	Hemoglobin	*188*	−0.294	<0.001	<0.001
CIDS-LOG	Preoperative eGFR	*185*	−0.196	0.008	0.009
CIDS-LOG	Preoperative creatinine	*185*	0.145	0.049	0.049
CIDS-LOG	Uric acid	*184*	0.316	<0.001	<0.001

Note: FDR adjustment was applied across the five prespecified markers within each CIDS definition. Abbreviations: CIDS, congestion-associated ionomic dysregulation score; eGFR, estimated glomerular filtration rate; FDR, false discovery rate; NT-proBNP, N-terminal pro-B-type natriuretic peptide.

**Table 4:** Multivariable association of CIDS with log1p(NT-proBNP).

Model	n	β	95% CI for β	exp(β)	95% CI for exp(β)	*p* Value	Adjusted R^2^
Raw CIDS Model 1: age + sex	*171*	0.712	0.447 to 0.976	2.04	1.56 to 2.65	<0.001	0.213
Raw CIDS Model 2: + preoperative eGFR + Hb	*170*	0.550	0.180 to 0.920	1.73	1.20 to 2.51	0.004	0.311
Raw CIDS Model 3: + heart failure + DM	*170*	0.480	0.134 to 0.827	1.62	1.14 to 2.29	0.007	0.382
Log-CIDS Model 1: age + sex	*171*	2.288	1.517 to 3.059	9.86	4.56 to 21.31	<0.001	0.249
Log-CIDS Model 2: + preoperative eGFR + Hb	*170*	1.865	0.977 to 2.753	6.46	2.66 to 15.69	<0.001	0.336
Log-CIDS Model 3: + heart failure + DM	*170*	1.598	0.698 to 2.497	4.94	2.01 to 12.15	<0.001	0.396

Note: Model 1 was adjusted for age and sex; Model 2 additionally included preoperative eGFR and hemoglobin; Model 3 additionally included heart failure and diabetes. exp(β) represents the multiplicative change in NT-proBNP + 1 per 1-unit increase in the score. Abbreviations: CIDS, congestion-associated ionomic dysregulation score; CI, confidence interval; eGFR, estimated glomerular filtration rate.

**Table 5:** Correlations of the four CIDS components with clinical markers.

Element	Outcome	n	Spearman ρ	*p* Value	FDR-Adjusted *p*
Cu	Hemoglobin	*188*	−0.009	0.908	0.908
Cu	NT-proBNP	*171*	0.158	0.038	0.077
Cu	Preoperative creatinine	*185*	−0.035	0.639	0.737
Cu	Preoperative eGFR	*185*	−0.064	0.388	0.776
Cu	Uric acid	*184*	−0.042	0.572	0.572
Fe	Hemoglobin	*188*	0.192	0.008	0.016
Fe	NT-proBNP	*171*	−0.068	0.373	0.498
Fe	Preoperative creatinine	*185*	−0.068	0.361	0.722
Fe	Preoperative eGFR	*185*	0.034	0.642	0.857
Fe	Uric acid	*184*	−0.137	0.064	0.255
Rb	Hemoglobin	*188*	0.195	0.007	0.016
Rb	NT-proBNP	*171*	−0.031	0.686	0.686
Rb	Preoperative creatinine	*185*	−0.025	0.737	0.737
Rb	Preoperative eGFR	*185*	0.010	0.894	0.894
Rb	Uric acid	*184*	−0.102	0.167	0.335
Sr	Hemoglobin	*188*	0.033	0.649	0.865
Sr	NT-proBNP	*171*	0.172	0.025	0.077
Sr	Preoperative creatinine	*185*	0.070	0.344	0.722
Sr	Preoperative eGFR	*185*	−0.094	0.201	0.776
Sr	Uric acid	*184*	0.074	0.319	0.425

Note: FDR adjustment was applied across the four prespecified elements within each clinical outcome. Abbreviations: CIDS, congestion-associated ionomic dysregulation score; eGFR, estimated glomerular filtration rate; FDR, false discovery rate; NT-proBNP, N-terminal pro-B-type natriuretic peptide.

**Table 6:** Exploratory associations of raw and log-transformed CIDS with cardiac phenotype and echocardiographic measures.

Model	n	Effect Estimate	95% CI	*p* Value	FDR-Adjusted *p* Value
CIDS-RAW: Heart failure (adjusted logistic model), OR	*190*	1.61	0.74 to 3.51	0.2275	0.3033
CIDS-RAW: E/e′ (adjusted linear model), β	*49*	1.04	0.19 to 1.89	0.0212	0.0849
CIDS-RAW: RVSP (adjusted linear model), β	*102*	1.35	−0.71 to 3.41	0.2034	0.3033
CIDS-RAW: LVEF (adjusted linear model), β	*151*	−0.74	−3.11 to 1.62	0.5389	0.5389
CIDS-LOG: Heart failure (adjusted logistic model), OR	*190*	11.91	1.66 to 85.26	0.0136	0.0545
CIDS-LOG: E/e′ (adjusted linear model), β	*49*	2.03	−0.69 to 4.75	0.1512	0.3023
CIDS-LOG: RVSP (adjusted linear model), β	*102*	2.62	−3.77 to 9.02	0.4231	0.4833
CIDS-LOG: LVEF (adjusted linear model), β	*151*	−2.75	−10.42 to 4.92	0.4833	0.4833

Note: Models were adjusted for age, sex, and referral group, with HC3 robust standard errors. Effect estimates are odds ratios for heart failure and regression coefficients for continuous outcomes. Benjamini–Hochberg FDR adjustment was applied across heart failure, E/e′, RVSP, and LVEF within each CIDS definition. Abbreviations: RVSP, estimated right ventricular systolic pressure; LVEF, left ventricular ejection fraction.

## Data Availability

Data supporting the analysis are available upon reasonable request by contacting the corresponding authors.

## Supplementary Materials

The following supporting information can be downloaded at https://www.mdpi.com/article/10.3390/molecules31203588/s1. Table S1: Incremental explanatory value of CIDS and its individual components for circulating NT-proBNP concentrations in the common complete-case sample (n = 170). Table S2: Internal bootstrap validation of the fully adjusted association between standardized raw CIDS and NT-proBNP. Table S3: Sensitivity analysis after exclusion of extreme NT-proBNP values. Table S4: Ordinal regression analysis of the association between CIDS and NYHA functional class. Table S5: Exploratory associations with angiographic vessel-disease burden (n = 53). Table S6: Exploratory perioperative renal outcomes. These analyses are statistically non-informative because only 40 participants and eight AKI events were available. Table S7: Permutation analyses comparing CIDS with alternative four-element composites. Table S8: Referral-pathway case-mix differences and stratified NT-proBNP models. Figure S1: Exploratory distribution of CIDS according to angiographic vessel-disease count. Figure S2A: Exploratory associations of raw CIDS with preoperative and postoperative renal measures. Figure S2B: Exploratory associations of log-transformed CIDS with preoperative and postoperative renal measures.

## Abbreviations

The following abbreviations are used in this manuscript:

AIC	akaike information criterion
AKI	acute kidney injury
CABG	coronary artery bypass grafting
CI	confidence interval
CIDS	congestion-associated ionomic dysregulation score
CKD	chronic kidney disease
CKD-EPI	Chronic Kidney Disease Epidemiology Collaboration
CRP	C-reactive protein
CV	coefficient of variation
DM	diabetes mellitus
E/e′	ratio of early transmitral flow velocity to early diastolic mitral annular velocity
eGFR	estimated glomerular filtration rate
FDR	false discovery rate
Hb	hemoglobin
HbA1c	glycated hemoglobin
HC3	heteroskedasticity-consistent covariance matrix estimator type 3
HDL	high-density lipoprotein
HF	heart failure
ICP-MS	inductively coupled plasma mass spectrometry
IQR	interquartile range
KDIGO	Kidney Disease: Improving Global Outcomes
LAVI	left atrial volume index
LDL	low-density lipoprotein
LOD	limit of detection
LVEF	left ventricular ejection fraction
NT-proBNP	N-terminal pro-B-type natriuretic peptide
OR	odds ratio
ORS	Octopole Reaction System
PCI	percutaneous coronary intervention
RF	radiofrequency
RVSP	right ventricular systolic pressure
SD	standard deviation
Element symbols:	
Ag	silver
Al	aluminum
As	arsenic
Ba	barium
Be	beryllium
Cd	cadmium
Co	cobalt
Cr	chromium
Cs	cesium
Cu	copper
Fe	iron
In	indium
K	potassium
Li	lithium
Mg	magnesium
Mn	manganese
Mo	molybdenum
Na	sodium
Ni	nickel
Pb	lead
Rb	rubidium
Sb	antimony
Se	selenium
Sr	strontium
Ti	titanium
Tl	thallium
U	uranium
V	vanadium
Zn	zinc
