# Supplementary material for: A Multielement Ionomic Dysregulation Pattern Is Associated with NT-proBNP and Cardiorenal Burden in Patients with Cardiovascular Disease
Source: Molecules. 2026 Oct 9;31(20):3588. doi: 10.3390/molecules31203588 (PMC13707558; doi:10.3390/molecules31203588)
Supplement: Supplementary file 1 [file molecules-31-03588-s001.zip › molecules-4572735-supplementary.pdf]

Supplementary Materials

A Multielement Ionomic Dysregulation Pattern Is Associated with NT-proBNP and Cardiorenal Burden in Patients with Cardiovascular Disease

Supplementary Table S1

Incremental explanatory value of CIDS and its individual components for circulating NT-proBNP concentrations in the common complete-case sample (n = 170).

Model	Added predictor(s)	Standardized β	95% CI	HC3 P	R^2	Adjusted R^2	ΔR^2	AIC
Clinical model	None	—	—	—	0.368	0.345	0.000	513.26
Clinical model + CIDS	CIDS, per 1 SD	0.276	0.077–0.475	0.007	0.407	0.382	0.039	504.32
Clinical model + Cu	Cu, per 1 SD	0.145	−0.020–0.310	0.087	0.380	0.353	0.012	511.95
Clinical model + Sr	Sr, per 1 SD	0.243	0.054–0.432	0.013	0.402	0.376	0.034	505.79
Clinical model + Rb	Rb, per 1 SD	0.078	−0.077–0.232	0.324	0.371	0.344	0.004	514.29
Clinical model + Fe	Fe, per 1 SD	0.027	−0.126–0.180	0.733	0.368	0.341	<0.001	515.14
Clinical model + all four elements	Cu + Sr + Rb + Fe	—	—	—	0.422	0.386	0.054	506.05

Note: All models used the identical complete-case sample. The clinical model included age, sex, preoperative eGFR, hemoglobin, heart failure, and diabetes. CIDS and individual elements were standardized using the full analytical cohort. HC3 inference used sandwich::vcovHC(type = "HC3") with lmtest::coeftest. The all-element model showed a significant joint HC3 Wald test in the prior analysis; the present table presents the directly reproduced model performance results.

Supplementary Table S2

Internal bootstrap validation of the fully adjusted association between standardized raw CIDS and NT-proBNP.

Validation parameter	Estimate
Complete-case sample size	170
Bootstrap resamples	2000
Apparent standardized CIDS coefficient, β	0.276
HC3 95% CI	0.077–0.475
HC3 P value	0.007
Bootstrap mean β	0.283
Bootstrap median β	0.286
Bootstrap SD of β	0.092
Bootstrap percentile 95% CI	0.099–0.454
Bootstrap resamples with β > 0	99.7%
Apparent R^2	0.407
Mean bootstrap training R^2	0.437
Mean test R^2 on original sample	0.371
Mean optimism in R^2	0.066
Optimism-corrected R^2	0.341

Note: Internal validation used 2000 nonparametric patient-level bootstrap resamples with replacement. The model included standardized raw CIDS, age, sex, preoperative eGFR, hemoglobin, heart failure, and diabetes, with log1p(NT-proBNP) as the dependent variable.

Supplementary Table S3

Sensitivity analysis after exclusion of extreme NT-proBNP values.

Analysis	n	CIDS effect	β	95% CI	HC3 P	R^2	Adjusted R^2
Primary model	170	Raw CIDS, per 1 unit	0.480	0.134–0.827	0.0066	0.407	0.382

Analysis	n	CIDS effect	β	95% CI	HC3 P	R^2	Adjusted R^2
Excluding upper 1%	168	Raw CIDS, per 1 unit	0.601	0.310–0.892	<0.001	0.382	0.355
Primary model	170	CIDS, per 1 SD	0.276	0.077–0.475	0.0073	0.407	0.382
Excluding upper 1%	168	CIDS, per 1 SD	0.345	0.178–0.512	<0.001	0.382	0.355

Supplementary Table S4

Ordinal regression analysis of the association between CIDS and NYHA functional class.

Model	n	Exposure	OR for higher NYHA category	95% CI	P
Model 1	187	CIDS, per 1 SD	1.70	1.27–2.28	<0.001
Model 2	185	CIDS, per 1 SD	1.01	0.71–1.43	0.956
Model 2	185	log1p(NT-proBNP)	52.88	25.11–111.34	<0.001

Supplementary Table S5

Exploratory associations with angiographic vessel-disease burden (n = 53).

Exposure	Outcome or model	Effect	95% CI	P	FDR-adjusted P
CIDS-RAW	Vessel-disease count (Spearman)	$\rho = -0.281$	—	0.041	0.074
CIDS-LOG	Vessel-disease count (Spearman)	$\rho = -0.268$	—	0.052	0.074
NT-proBNP	Vessel-disease count (Spearman)	$\rho = -0.276$	—	0.076	0.076
CIDS-RAW	Age- and sex-adjusted linear model	$\beta = -0.264$	−0.978–0.449	0.460	NA
CIDS-RAW	Multivessel disease logistic model	OR = 0.40	0.04–4.25	0.446	NA
CIDS-RAW	Three-vessel disease logistic model	OR = 0.55	0.05–6.18	0.631	NA

Supplementary Table S6

Exploratory perioperative renal outcomes. These analyses are statistically non-informative because only 40 participants and eight AKI events were available.

Score	Renal marker or model	n	Effect	P	FDR-adjusted P
CIDS-RAW	Preoperative creatinine	185	$\rho = 0.123$	0.096	0.337
CIDS-RAW	Preoperative eGFR	185	$\rho = -0.191$	0.009	0.065
CIDS-RAW	Postoperative peak creatinine	40	$\rho = 0.077$	0.636	0.738
CIDS-RAW	Postoperative calculated eGFR	40	$\rho = -0.132$	0.416	0.738
CIDS-RAW	Change in eGFR	40	$\rho = -0.055$	0.738	0.738
CIDS-RAW	Change in creatinine	40	$\rho = 0.065$	0.690	0.738
CIDS-RAW	KDIGO AKI stage	40	$\rho = 0.060$	0.712	0.738
CIDS-LOG	Preoperative creatinine	185	$\rho = 0.145$	0.049	0.173
CIDS-LOG	Preoperative eGFR	185	$\rho = -0.196$	0.008	0.053
CIDS-LOG	Postoperative peak creatinine	40	$\rho = 0.115$	0.479	0.779
CIDS-LOG	Postoperative calculated eGFR	40	$\rho = -0.159$	0.327	0.763
CIDS-LOG	Change in eGFR	40	$\rho = -0.046$	0.779	0.779
CIDS-LOG	Change in creatinine	40	$\rho = 0.075$	0.648	0.779
CIDS-LOG	KDIGO AKI stage	40	$\rho = 0.062$	0.703	0.779
CIDS-RAW	Postoperative AKI, unadjusted	40	OR = 1.82	0.679	NA
CIDS-RAW	Postoperative AKI, adjusted for preoperative eGFR	40	OR = 1.91	0.667	NA

The AKI odds-ratio confidence intervals were 0.11–31.54 and 0.10–36.14, respectively. These estimates are retained only for transparency and should not be interpreted as evidence for or against an association.

Supplementary Table S7

Permutation analyses comparing CIDS with alternative four-element composites.

Analysis	Null iterations	Observed ΔR²	Null median ΔR²	Null 95th percentile	Empirical P
Observed CIDS	—	0.0394	—	—	—
All 16 equal-weight sign patterns	16	0.0394	0.0147	0.0394	0.176
Random normalized weights	10,000	0.0394	0.0107	0.0414	0.063

Note: Every candidate composite was added to the same complete-case clinical reference model. Random weight vectors were normalized to the Euclidean length of the observed CIDS weight vector. Empirical P = (1 + number of null ΔR^2 values ≥ observed ΔR^2)/(1 + number of null iterations). The observed score did not clearly exceed either null distribution.

Supplementary Table S8

Referral-pathway case-mix differences and stratified NT-proBNP models.

Panel A. Available case-mix variables

Variable	Referral group 0	Referral group 1	SMD
Age	66.85	65.82	0.111
Male sex	55.1%	79.7%	−0.545
Preoperative eGFR	81.76	84.63	−0.138
Hemoglobin	8.72	8.85	−0.134
Heart failure	26.3%	16.7%	0.236
Diabetes	34.7%	37.5%	−0.057
NT-proBNP	911.33	405.60	0.175
Cu	581.54	582.63	−0.003
Sr	19.99	21.11	−0.060
Rb	142.31	137.50	0.047
Fe	810.04	566.60	0.353

Panel B. HC3-robust adjusted models

Model	n	β per 1-SD CIDS	95% CI	P
Pooled primary	170	0.276	0.077–0.475	0.007
Pooled + referral group	170	0.256	0.049–0.463	0.016
CIDS × referral-group interaction	170	0.275	−0.117–0.667	0.171
Referral group 0	111	0.179	−0.085–0.443	0.187
Referral group 1	59	0.569	0.112–1.026	0.018

Note: Stratified estimates differed in magnitude. The non-significant interaction should not be interpreted as proof of homogeneous effects because power was limited and case mix differed between pathways.

Supplementary Figure S1

Exploratory distribution of CIDS according to angiographic vessel-disease count.

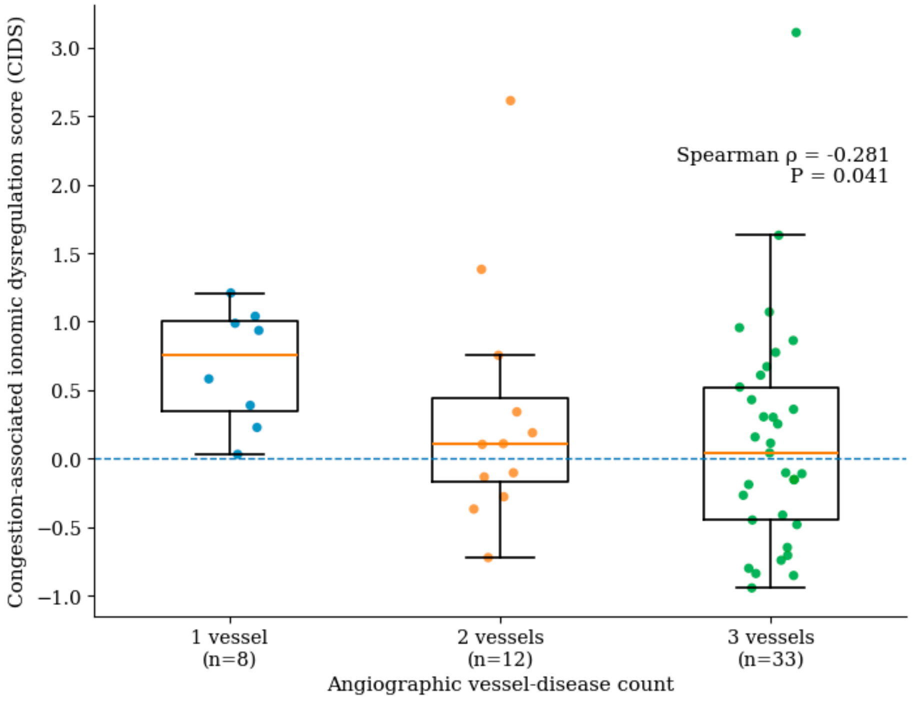

Box-and-whisker plots show the distribution of CIDS across angiographic vessel-disease count. This analysis was exploratory.

Supplementary Figure S2A

Exploratory associations of raw CIDS with preoperative and postoperative renal measures.

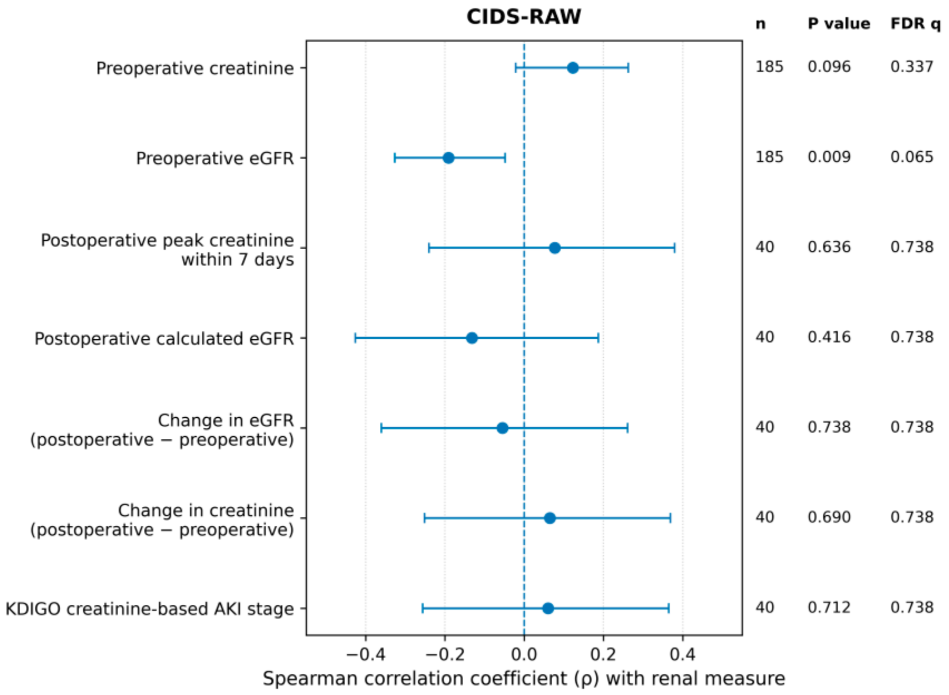

Postoperative estimates were restricted to 40 participants and are statistically non-informative.

Supplementary Figure S2B

Exploratory associations of log-transformed CIDS with preoperative and postoperative renal measures.

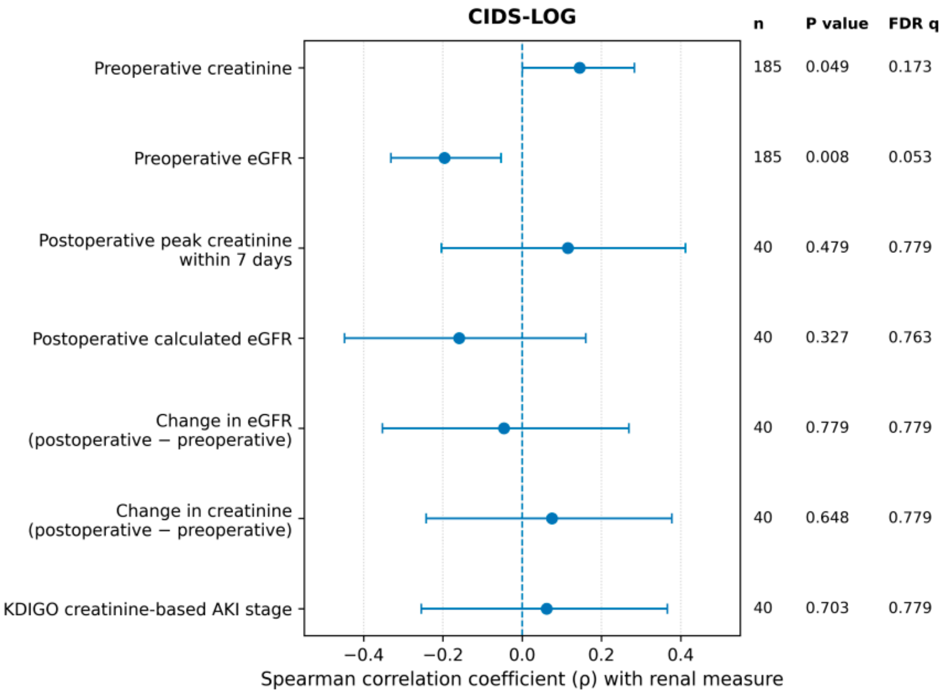

Postoperative estimates were restricted to 40 participants and are statistically non-informative.
